# Lack of Group Support and Burnout Syndrome in Workers of the State Security Forces and Corps: Moderating Role of Neuroticism

**DOI:** 10.3390/medicina55090536

**Published:** 2019-08-26

**Authors:** Jesús Farfán, Marta Peña, Gabriela Topa

**Affiliations:** 1 Health Psychology Program, International School of Doctorate, National Distance Education University (UNED), Juan del Rosal 10, 28040 Madrid, Spain; 2 Department of Social and Organizational Psychology, National Distance Education University (UNED), Juan del Rosal 10, 28040 Madrid, Spain; 3 Facultad de Ciencias de la Salud, Universidad Politécnica y Artística del Paraguay, Asunción 1628, Paraguay

**Keywords:** burnout, neuroticism, group support, personality traits, emotional exhaustion

## Abstract

*Background and Objectives*: This research analyzes the relationship between the lack of group support and burnout syndrome in workers of the State Security Forces and Corps, considering the role of personality traits in this relationship. In particular, it is hypothesized that neuroticism will moderate this relationship. *Materials and Methods*: Participants were 237 workers from the State Security Forces and Corps dedicated to tasks of citizen security. *Results*: The results show that neuroticism moderates the relationship between lack of group support and the three components of the burnout syndrome: emotional exhaustion, depersonalization, and personal fulfillment. *Conclusions*: The findings are discussed, suggesting intervention strategies for the improvement of the agents’ personal well-being.

## 1. Introduction

Stress is the second most common health problem at work and one of the first threats to the productivity of organizations. It is estimated that, in the European Union alone, between 50% and 60% of labor casualties are caused by stress. The Spanish Association of Occupational Medicine Specialists stated that, in Europe, 40 million workers could be affected by stress in the year 2012. The economic cost of this problem is at least 20 billion euros per year. Despite the enormous extension of this problem, only 26% of European companies take measures aimed at reducing the incidence of work stress [1]. 

From the approach of psychology of sustainability and sustainable development [2,3,4], it is emphasized that healthy organizations are those in which the work setting is characterized by favoring employees’ well-being while achieving organizational objectives. The work environment that lacks cooperative behaviors and peer collaboration and support has long been recognized as a clear predictor of work stress [5]. However, the literature also recognizes personal characteristics as moderators in the relationship between the antecedents of the work environment and the employees’ attitudinal and behavioral outcomes [6]. The continuous stress situations that police work can produce could cause burnout in the agent and, consequently, cause poor actions that put people’s lives at risk. For this reason, it is very important to know those factors that can favor the appearance of burnout. In particular, it has been found that personality plays an important role in the probability of occurrence of stressful events [7], and in the manner in which these events are perceived and managed [8]. Among the personality traits that are associated with a worse psychological adaptation and are linked to a greater number of health problems is neuroticism, which is associated with more negative emotions in general [9,10,11,12,13]. Some authors have pointed out that stress may be more related to neuroticism than to other variables of the working context [14]. In summary, both the characteristics of the working environment, on the one hand, and the worker’s personality traits, on the other, seem to influence job stress and contribute to an increase in costs for the company.

Therefore, the objective of this research is to analyze the relationships between the lack of working group support and stress, taking into consideration the potential moderator role of the employee’s neuroticism. Thus, the present study aims, firstly, to provide information about individual characteristics of those workers who are more resistant to stress. It could help to improve the organizational strategies of personnel recruitment, favoring processes of selection oriented to identifying candidates that would better adapt themselves under unfavorable working conditions. Secondly, this article aims to contribute to the promotion of workers’ welfare by facilitating the design of interventions that increase group support. Third, although personality traits seem relatively stable over time, it is also often accepted that people can be guided and helped to develop strengths that allow them to compensate, even if only partially, the negative impact of neuroticism on their well-being. 

### 1.1. Continued Occupational Stress: Burnout 

Members of the State Security Forces and Corps regularly face situations that generate a high level of stress, which seems to be the cause of a high number of medical casualties within this group. According to data provided by the Ministry of Home Affairs, there seems to be an increase in the percentage of agents who took sick leave due to psychic causes: 0.38% in 2006 and 2005, 0.4% in 2007, and 0.5% in 2008 [15]. Stress is defined by the World Health Organization as “the set of physiological reactions that prepares an organism for action” [16]. In response to this definition, one might think that the effects of stress are beneficial, but they become a real problem when the individual perceives circumstances as threatening, the danger is exaggerated, and these perceptions generate nonadaptive defense reactions. 

The response of stress is not harmful itself; on the contrary, it is an adaptive reaction that has allowed species to survive. It is very useful—one could say vital—to respond to threatening situations. However, when this reaction appears in a very recurrent, intense, or long-lasting way, it can produce a waste of resources, favoring the emergence of deteriorated performance, physical and emotional discomfort, and diseases, among other pathologies associated with stress [17,18]. Burnout is a psychosocial phenomenon that has three large dimensions. First, emotional exhaustion characterized by lack of energy, enthusiasm, and a feeling of lack of resources; second, depersonalization, a characteristic that is evidenced by treating the organization, customers, and colleagues as objects; and third, lack of professional achievement, a behavioral phenomenon evidenced by a tendency of the worker to self-assess negatively, leading professionals to feel dissatisfied with their performance at work [19]. In this way, high scores in emotional fatigue and depersonalization, as well as low scores in personal achievement, indicate higher levels of burnout in an individual [20].

When job stress is persistent, the burnout syndrome may arise [21]. Burnout consists of a psychological state of exhaustion and a negative and insensitive attitude towards the recipients of the services, associated with amotivation, feelings of failure, and diminished performance. It is frequent in professionals who carry out their work in contact with other people (patients, clients, students, users). Maslach and Jackson [22] consider that it is an inadequate response to a chronic stressor characterized by three dimensions: exhaustion or emotional exhaustion, depersonalization or dehumanization, and lack or decrease in personal accomplishment at work. 

### 1.2. Burnout and Police Work: The Importance of the Work-Group Support 

The characteristics of the work environment are frequently pointed to as antecedents of burnout [23]. In addition to working conditions, such as noise, heat, moisture, vibration, working with toxic or hazardous substances, the characteristics of the work environment, such as the leadership style of supervisors and the support of peers, have been repeatedly indicated as antecedents of burnout [24]. Moreover, in almost all work settings, the lack of work-group support has been considered a precursor to burnout. As mentioned by Jackson, Schwab, and Schuler [25], the absence of a support group can lead to exhaustion at work. An effective support group includes people who provide emotional comfort, confront the individual in a friendly way when his or her behavior is inappropriate, provide technical support in work-related areas, propose technical challenges that foster individual growth, serve as active listeners, and share similar values, beliefs, and perceptions of reality. Whether it is considered a direct antecedent [26] or a mediator of the influence of more remote antecedents [27,28], the role of group support as a negative antecedent of stress seems clearly established. The research carried out by Kobasa [29] demonstrated that social support is an important resource that facilitates individuals’ psychological, physical, and general well-being.

Police work is considered stressful and dangerous because of exposure to confrontation, violence, and traumatic incidents, including the possibility of being injured or killed [30,31]. In this sense, Cannizo and Liu [32] frame police activity among the service professions that demand both emotional stability and the skill to make quick decisions. According to these authors, the imbalance between psychological and emotional demands and the absence or scarcity of positive and pleasant interactions would lead to the perception of stress by police agents. While private companies insist on the importance of the organization of work and of job content for productivity, in police work, the workers’ participation in decisions about the organization of the work is less flexible. Coupled with all this, certain aspects of the work, such as high demand and low control, shift work, and frequent contact with citizens, define this profession as high-stress. On another hand, the exposure of the police agent, not only as a target of danger or injury, but as a witness to the suffering of others, is a factor that generates stress over time. Finally, the perception that no positive aspects derive from their work could lead them to do only what they are asked, avoiding any personal contribution or effort [33].

### 1.3. Personality as Moderator of the Burnout-Outcomes Relationship

The presence of potentially stressful situations does not affect all individuals in the same way. According to the available evidence, among other reasons, this is because personality and stress are closely related [34]. Firstly, personality traits are linked to choosing or avoiding environments associated with specific stressors. Secondly, personality is associated with the way one interprets a stressful situation and evaluates one’s skills and capacities to confront or avoid the situation. Thirdly, personality is associated with differences in the intensity of the response to a stressful situation and, finally, personality is also linked to the coping strategies employed by the individual when facing a stressful situation [35,36,37]. In particular, some studies have reported considerable consistency in the strategies used to cope with stressful situations based on personality traits, such as neuroticism, regardless of situational factors [35]. Different authors suggest that the analysis of the relationship between stressful situations and people’s well-being as a function of personality traits will help to understand and explain why certain personality factors are related to the level of adaptation of the person undergoing stressful situations [36].

In the study of personality in the field of health, the five-factor model [37] currently represents one of the most consolidated paradigms. This model has been used in various transcultural studies that have confirmed its structural strength through factor analysis [38]. The big five model proposes the existence of five major factors, or basic personality dimensions, with which one can describe a person. These traits are relatively stable tendencies that present characteristic ways of behaving or responding to a varied number of situations and are manifested specifically in attitudes, habits, patterns of interpersonal interaction, motives, and interests. Costa and McCrae consider five personality factors: “neuroticism, extraversion, openness to experience, agreeableness (or kindness), and conscientiousness”. Neuroticism is the general tendency to experience negative emotions such as fear, melancholy, shame, anger, guilt and disgust, to have irrational ideas, to be less able to control one’s impulses, and to cope with stress worse than other people. It is the opposite of emotional stability and implies the tendency to experience negative feelings and to have difficulties in situations that generate stress. 

Neuroticism seems to favor the perception of stimuli as more threatening and anxiety-provoking, thus influencing a large number of aspects of working and personal life. Some research indicates that neuroticism is negatively related to the perception of life satisfaction [39]. Neuroticism correlates positively with hostile reactions to stressful events and is intimately related to nonadaptive coping responses [40]. In general, it coincides with measures such as chronic anxiety, negative affect, depression, hostility, or emotional vulnerability. When environmental pressures are high, people with low levels of neuroticism achieve better performance than people with high neuroticism. 

In the study of Hart, Wearing, and Headey [13], it was concluded that personality traits are the determinants with the greatest impact on police agents’ psychological distress and welfare, and neuroticism was shown to be a significant predictor of perceived quality of life. Moreover, even though extraversion positively influenced perceived quality of life, it did not have the same impact as neuroticism. Neuroticism and extraversion predicted the use of stress-coping strategies and the types of experiences (problematic or motivating) encountered at work. With regard to the capacity of the big five model to predict success in the profession, the variable neuroticism predicts low job satisfaction and an increase in reactivity when facing threats, and the avoidance of possible harm [41].

With regard to the capacity of the big five model to predict professional success, according to Olmedo [42], the investigations of Carver, Sutton, and Scheier [43] conclude that neuroticism predicts higher job and organization turnover. Zellars, Perrewé, and Hochwarte [44] applying the Maslach burnout inventory (MBI) and the big-five questionnaire (BFQ) to hospital staff, found positive relationships between the three factors of burnout syndrome and personality traits. Following this line of research, Ghorpade, Lackritz, and Singh [12] carried out a regressive analysis on the responses of 265 university professors to the MBI and the BFQ, finding that depersonalization is negatively related to agreeableness and neuroticism. Finally, personal realization has a negative association with neuroticism [45].

In short, there is a growing body of empirical evidence that supports neuroticism’s relationships with stress responses and other indicators of well-being and health, whereas this evidence is more limited for other personality traits. Therefore, in the present study, we have deepened the analysis of the moderator role of neuroticism in the relation between the work environment and workers’ burnout.

Based on the literature reviewed herein, this research analyzes the relationships between the lack of work-group support and burnout, in its three dimensions: emotional exhaustion, depersonalization, and personal fulfillment. Personality characteristics, neuroticism, and extraversion are proposed as moderators of the relationship between lack of support and burnout. The following hypotheses are established:

**Hypotheses** **1** **(H1).** *“The lack of group support predicts burnout, and this relationship is moderated by neuroticism.”*

**Hypotheses** **1A** **(H1a).** *“Neuroticism moderates the relationship between the lack of group support and emotional exhaustion.”*

**Hypotheses** **1B** **(H1b).** *“Neuroticism moderates the relationship between the lack of group support and depersonalization.”*

**Hypotheses** **1C** **(H1c).** *“Neuroticism moderates the relationship between the lack of group support and personal fulfillment.”*

## 2. Method

### 2.1. Participants

The investigation was carried out on a voluntary sample of 237 people, 75.95% men and 24.05% women, with a mean age of 37.72 years, belonging to the State Security Forces and Corps in Spain, in a situation of active service, who habitually carry out tasks of citizen security. Concerning participants’ marital situation, 35.4% were single, 27.8% were married, 10.5% were divorced, and 26.2% were living as a couple. Regarding education, 49.5% had completed secondary education, 37.1% had high school studies, and 13.5% had university studies. The average age of entering the organization was 23.2 years and mean job tenure was 14.04 years. Concerning their families, 48.5% of the participants had no children, 30.4% had only one child, and 20.4% had two or more children. Most participants (76.8%) were working in shifts. 

### 2.2. Instruments

“Neuroticism”: This was evaluated with the revised NEO personality inventory (NEO PI-R), which offers a 240-item measure of the five main dimensions of the adult personality, 48 items in each dimension [37]: neuroticism, extraversion, openness, agreeableness, and conscientiousness. It is rated on a 5-point Likert scale (depending on the degree of agreement with the statement). Although the entire questionnaire was completed, the relevant answers in this research were those related to neuroticism (N). In this study, the Cronbach reliability alpha was *α* = 0.87 for neuroticism. 

“Burnout”: The instrument used was the Maslach burnout inventory-general survey (MBI-GS) [22], in the adaptation to the Spanish population by Gil-Monte [46], which evaluates three dimensions: emotional exhaustion (9 items), depersonalization (5 items), and personal fulfillment (8 items) [47]. In the present study, the Cronbach alpha values were higher than 0.70 for all dimensions. Emotional exhaustion α = 0.83, depersonalization α = 0.74, and professional fulfillment α = 0.85. Usually, whereas in the subscales of emotional exhaustion and depersonalization, high scores correspond to high feelings of burnout, in the subscale of personal fulfillment at work, low scores correspond to high feelings of burnout [47]. 

“Lack of work-group support”: This was assessed using the work-group support subscale of the World Health Organization Questionnaire (ILO-WHO) [48]. The subscale consists of 3 items: “My coworkers do not support my professional goals,” “My coworkers do not offer me enough protection from the unfair work demands of the bosses,” and “My coworkers do not give me enough help when I need it.” Responses were rated on a 7-point Likert-type scale, ranging from 1 (“strongly disagree”) and 7 (“strongly agree”). Despite the brevity of the scale, the Cronbach alpha value for the present study was adequate (α = 0.75).

### 2.3. Procedure

The ethical committee of the UNED approved the project under the protocol number 07172018 (approval date: 17th July 2018). Participants were allocated into 20 groups of about 10–15 individuals per group, and each group completed all the questionnaires at the same time in a room specifically prepared for this aim. Participants were informed of the objective of the investigation and of the conditions of anonymity and voluntariness of their participation. Those who agreed to participate were invited to complete the questionnaire on an individual basis after signing the informed consent. One of the members of the research team was present while the participants completed the survey to resolve doubts and answer questions. The completed paper-format questionnaires were delivered in a closed envelope to the member of the research team.

## 3. Results

Correlational analysis (Table 1) shows that the lack of group support correlated positively and significantly with emotional exhaustion and depersonalization and negatively and significantly with personal fulfillment. Emotional exhaustion and depersonalization both correlated negatively and significantly with personal fulfillment and were significantly and positively correlated with each other. These results preliminarily confirm the proposed hypothesis. 

**Figure 1:**
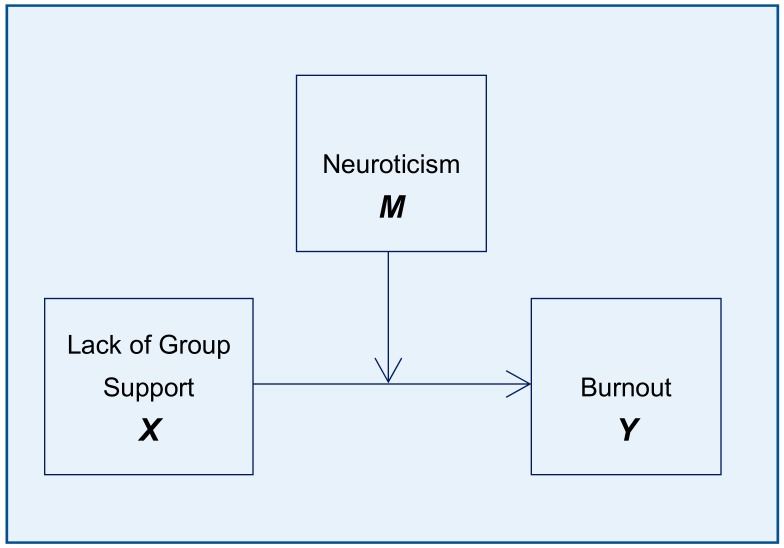
Moderation model depicting neuroticism as a regulation factor of the effect of lack of group support over burnout.

With regard to the macro analysis with Hayes’ (2013) Process, the simple moderation model (Model 1) was used with the quantitative moderator variable (Figure 1). In all the analyses, the number of bootstrap samples used was 10,000 with a 95% confidence level. In all cases, three coefficients were obtained: The b2 coefficient estimates the main effect of the moderator neuroticism (M) on the criterion, burnout (Y); the b1 coefficient estimates the effect of the predictor, lack of group support (X), on the criterion, burnout (Y); and the b3 coefficient estimates the effect of the interaction of the predictors on the criterion, burnout (Y).

Three levels were considered in the moderator neuroticism: low neuroticism (40.63), medium neuroticism (49.73), and high neuroticism (58.84).

In relation to Hypothesis H1a, the results indicate that the three coefficients obtained in the regression analysis were significant. The effect of neuroticism was significant (B [Neuroticism] = 0.84, *p* < 0.0008; [95%LLCI = 0.51/ULCI = 1.17]), the effect of X was significant (B [Lack of group support] = 5.52, *p* < 0.000; [95%LLCI = 3.49/ULCI = 7.55]), and the interaction was also significant (B [Lack of group support × Neuroticism]) = −0.07, *p* < 0.0003; [95% LLCI = −0.11/ULCI = −0.03]). A total of 43% of the variance was explained by the model, and a 0.03% increase was due to the interaction (*p* = 0.0003). 

**Figure 2:**
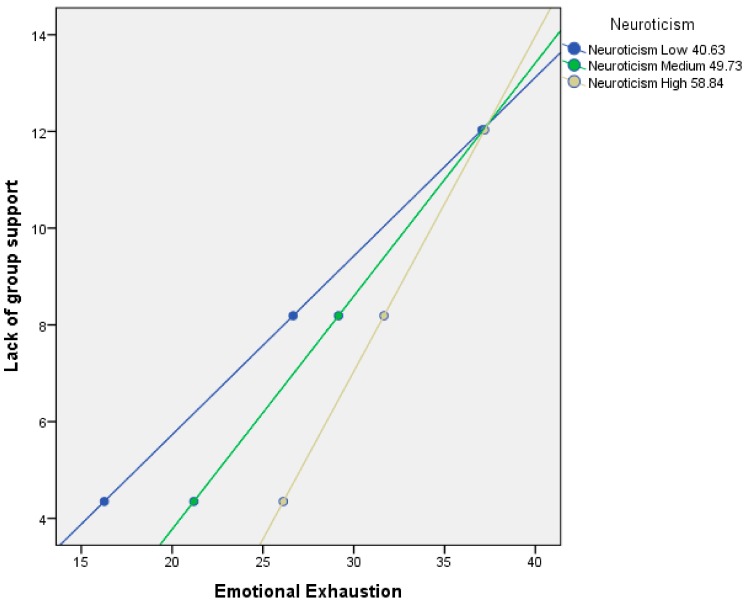
Conditional effect of the lack of group support on emotional exhaustion as a function of the moderation of neuroticism.

When analyzing the conditional effect of X on Y, the effect of emotional exhaustion on the lack of group support was statistically significant in all three levels of neuroticism, that is, in people with low (X→YIM = 40.63 = 2.70, *p* < 0.000; [95%LLCI = 2.11/ULCI = 3.30]), medium (X→YIM = 49.73 = 2.07, *p* < 0.001; [95%LLCI = 1.71/ULCI = 2.43]), and high neuroticism (X→YIM = 58.84 = 1.44, *p* < 0.001; [95%LLCI = 1.06/ULCI = 1.81]) (Figure 2).

In relation to hypothesis H1b, the results showed that all three coefficients were significant. The effect of neuroticism was significant (B [Neuroticism] = 0.31, *p* < 0.0002; [95%LLCI = 0.15/ULCI = 0.47]), the effect of X was significant (B [Lack of group support] = 2.18, *p* < 0.001; [95%LLCI = 1.18/ULCI = 3.17]) and the interaction (B [Lack of support × Neuroticism]) was significant = −0.03, *p* < 0.005; [95%LLCI = −0.04/ULCI = −0.007]). A total of 36.6% of the variance was explained by the model, and the increase in variance due to the interaction was 0.02% (*p* = 0.005).

**Figure 3:**
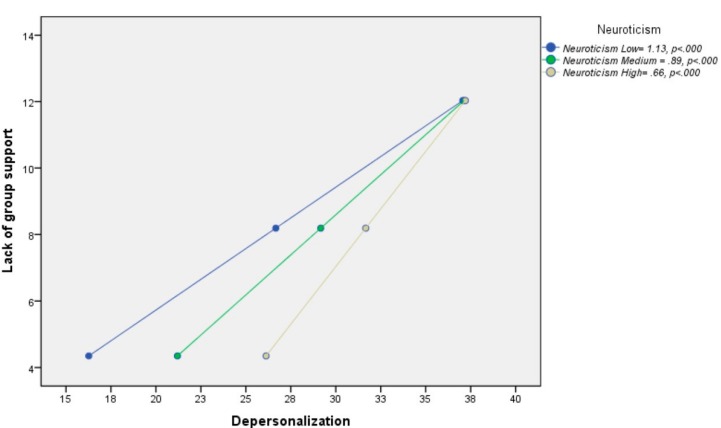
Conditional effect of lack of group support on depersonalization as a function of the moderation of neuroticism.

Regarding the conditional effect of X on Y, it was found that the effect of lack of support on depersonalization was statistically significant among participants with low (X→YIM = 40.63 = 1.13, *p* < 0.001; [95%LLCI = 0.83/ULCI = 1.42]), medium (X→YIM = 49.73 = 0.89, *p* < 0.001; [95%LLCI = 0.71/ULCI = 1.07]), and high neuroticism (X→YIM = 58.84 = 0.66, *p* < 0.001; [95%LLCI = 0.47/ULCI = 0.84]) (Figure 3).

In relation to hypothesis H1c, all three coefficients were significant. In other words, the effect of neuroticism was significant (B [Neuroticism] = −0.60, *p* < 0.0001; [95%LLCI = −0.88/ULCI = −0.30]), the effect of X was significant (B [Lack of support] = −3.67, *p* < 0.001; [95%LLCI = −5.44/ULCI = −1.89]), and the interaction was significant (B [Lack of support × Neuroticism] = 0.04, *p* < 0.01; [95%LLCI = 0.007/ULCI = 0.07]). The percentage of variance explained by the complete model was 42.5%, and the increase due to the interaction was 0.01% (*p* = 0.016). 

**Figure 4:**
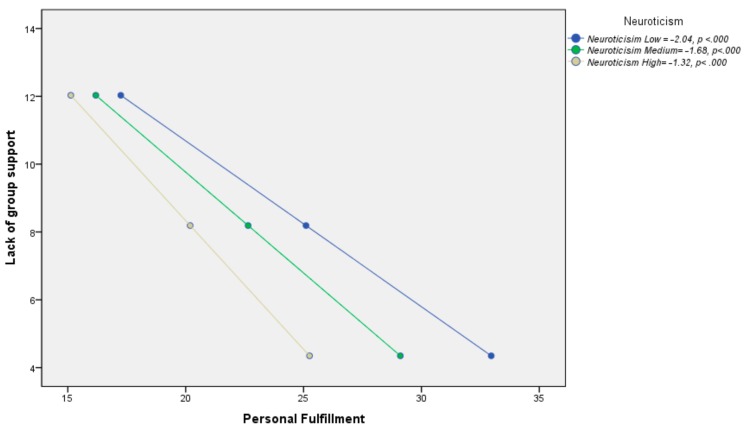
Conditional effect of the lack of group support on personal fulfillment as a function of the moderation of neuroticism.

When analyzing the conditional effect of X on Y in the different values of the moderator, neuroticism, it was again found that the effect of lack of support on personal fulfillment was statistically significant for low (X→YIM = 40.63 = −2.04, *p* < 0.000; [95%LLCI = −2.56/ULCI = −1.52]), medium (X→YIM = 49.73 = −1.68, *p* < 0.000; [95%LLCI = −1.99/ULCI = −1.36]), and high neuroticism (X→YIM = 58.84 = −1.32, *p* < 0.000; [95%LLCI = −1.64/ULCI = −0.99]). However, in this case, the effect of the predictor variable, lack of support, on the criterion, burnout, was negative, as mentioned in the correlational analysis (Figure 4).

## 4. Discussion

The objective of our research was to analyze the role of neuroticism in the relationship between the lack of work-group support and the burnout syndrome in subjects who are engaged in a potentially stressful profession, such as that performed by members of the State Security Forces and Corps. The results confirm the proposed hypotheses, because the effect of the interaction between the lack of group support and neuroticism was significant for emotional exhaustion, depersonalization, and personal fulfillment. On another hand, when analyzing the conditional effects of lack of group support on emotional exhaustion, depersonalization, and personal fulfillment, these effects were significant for all levels of neuroticism. In emotional exhaustion, when the lack of group support is low, participants with lower neuroticism show less exhaustion, whereas those with high scores in neuroticism show more emotional exhaustion. However, when the lack of group support increases, the scores of the three groups are similar, with emotional exhaustion being higher for all levels of neuroticism. In the analysis of the relationship between the lack of group support and depersonalization, a similar result was observed. When the lack of group support is low, participants with low neuroticism show less depersonalization, but when the lack of support increases, the depersonalization scores of the three groups resemble each other. As for personal fulfillment, when the lack of group support is low, participants with high neuroticism show less personal fulfillment than those of the other two categories. When the lack of support increases, the scores of personal fulfillment of all three groups of neuroticism are similar, but the personal fulfillment of individuals scoring high in neuroticism is lower. 

These results are in no way conclusive, but the trends suggest that people who show greater emotional maladjustment (e.g., they are more unstable emotionally) have less emotional and personal resources, as well as a tendency to cease considering users as people, treating them as if they were objects. Moreover, more emotionally unstable people tend to feel less effective and fulfilled in their work. The present findings are in line with the meta-analysis of Alarcon, Eschleman, and Bowling [49], which showed the negative relationship between emotional stability and emotional exhaustion and between emotional stability and depersonalization. Although the authors of the review did not propose specific hypotheses for neuroticism, their findings marked a line of research that was followed by other works. Specifically, Lue, Chen, Wang, and Chen [50] verified the relationship between neuroticism and burnout among first-year medical residents in a nationwide study in Taiwan. These works coincide with the proposal of Bakker and collaborators [6], according to which personality characteristics affect the perception of job demands and resources. In particular, people high in neuroticism reported higher job demands.

Therefore, with regard to personality factors, the results indicate that high neuroticism poses a higher risk factor for the onset of burnout, with group support acting as a protective factor. These results are consistent with the findings obtained by Hart et al. [13], which report significant correlations between neuroticism and job stress. They also coincide with the findings of other authors [40,51], which conclude that neuroticism is closely related to nonadaptive coping responses, and with results in which significant and positive correlations were obtained between emotional exhaustion and depersonalization [52,53] and negative correlations with personal fulfillment at work [44]. However, in our research, personal fulfillment was not significant.

The results obtained in our research indicate a significant relationship between some of the big five personality factors, in particular, neuroticism, and the three dimensions of burnout. Our findings are in line with suggestions made by Scheier and Carver [54], which defend that neuroticism predicts low psychological and physical wellbeing and is linked to an increase in reactivity to threats and the avoidance of possible harm, associated with an increase of stress levels. In the same line are the results obtained by Zellars et al. [44] with hospital staff, and with the results of Ghorpade et al. [12] in teaching staff, and other researchers with university students [55]. Although these results do not allow for the establishment of a causal relationship between the variables, they do seem to confirm that, in this collective of workers dedicated to police tasks, the traits of neuroticism and perceived occupational stress are related to each other. That is, there is a positive and significant correlation between neuroticism and burnout, as recent studies with police officers from other countries suggested [56]. 

Studies with samples of anesthetists also provide coincident evidence with the present study. Neuroticism consistently influences the appraisal of stress and coping and, consequently, the development of burnout [57]. In the same line, other works have shown that neuroticism has direct effects on emotional exhaustion and depersonalization among nurses. The authors suggest that intervention programs should focus on helping nurses to increase their emotion regulation so that they will be able to successfully prevent and cope with future burnout situations [11]. These findings are also in line with those of studies on Dutch medical residents’ personality traits and burnout [58]. Neuroticism was significantly related to the burnout of residents in all specialties, but this relationship was stronger in residents of specialties such as surgery or support specialists (anesthetists, nuclear medicine, radiotherapy, among others) than in residents of general medicine. These findings were significant even when controlling for gender and some characteristics of the working environment, such as autonomy and satisfaction with the relationship between work and private life.

## 5. Conclusions

Limitations and lines of future research

Some important limitations must be acknowledged in this research. First, a small sample of subjects was used, which implies that it is not representative of the population, and this prevents generalizing the results. In addition, the entire sample was from the same police organization. Although this allows for the analysis of a more homogeneous situation, it would be of great interest to extend the study to other police bodies, such as local and regional police. On the other hand, sporadically, the answers offered in the self-reports may be affected by the respondents’ faking behavior, distorting the results. The NEO PI-R inventory has reliable scales to prevent this problem; however, these scales are not available in the MBI questionnaire or in the ILO-WHO labor stress questionnaire [59]. This research did not take social desirability into account, which affects the applied surveys. Although the results obtained by participants in the NEO PI-R inventory did not invalidate the tests, in future investigations we recommend applying sincerity scales to eliminate participants who distort their responses. In addition, among the potential moderators of the relationship between the work environment and burnout, only neuroticism was considered in this study. Among the personal variables that could have been analyzed are extraversion, and personal strengths, such as resilience, optimism, or executive functions [10]. 

Applicants for entry into police forces must pass an exhaustive selection process. In relation to their psychological capacity, they must perform different intelligence and personality questionnaires, as well as a personal interview conducted by several psychologists. The interviewers use the results of these personality questionnaires to explore the adaptation to the stress of the candidates and select those that are predictably better to adapt to the high levels of stress to which they will be exposed. It is very useful to know what personality factors can affect their future task performance. Among other reasons, this performance has very relevant consequences for citizens’ well-being, for the safety of social groups, and for the professional promotion and career development of the police themselves. The present findings provide information on the influence of personality factors in the tendency to suffer dysfunctional symptoms from stressors in the working environment. As studies in other professional fields recently suggested, personality traits would directly predict burnout [60]. This information can help to predict a worker’s performance when faced with a specific task that may generate high levels of stress. In addition, these findings suggest the desirability of designing intervention programs that reinforce personal strengths to favor effective coping of stressful situations. Personal strengths such as resilience, optimism, or hope are traits and competencies that have a protective effect on people, they are oriented to increasing the well-being of individuals and their communities, and they can be applied in a wide range of situations [61].

## Figures and Tables

**Table 1:** Pearson correlation matrix among research variables.

Variables	Mean	SD	Range	1	2	3	4
1. Lack of group support	8.19	3.84	3–21				
2. Emotional exhaustion	28.51	12.49	7–52	0.603 **			
3. Depersonalization	18.50	5.77	6–29	0.566 **	0.826 **		
4. Personal fulfillment	23.02	10.79	4–46	−0.601 **	−0.821 **	−0.800 **	
5. Neuroticism	49.73	9.10	27–70	0.270 **	0.353 **	0.303 **	−0.376 **

** *p* < 0.01.
